# Ligand-based virtual screening interface between PyMOL and LiSiCA

**DOI:** 10.1186/s13321-016-0157-z

**Published:** 2016-09-07

**Authors:** Athira Dilip, Samo Lešnik, Tanja Štular, Dušanka Janežič, Janez Konc

**Affiliations:** 1 National Institute of Chemistry, Hajdrihova 19, 1000 Ljubljana, Slovenia; 2 Faculty of Mathematics, Natural Sciences and Information Technologies, University of Primorska, Glagoljaška 8, 6000 Koper, Slovenia; 3 Laboratory for Physical Chemistry and Thermodynamics, Faculty of Chemistry and Chemical Technology, University of Maribor, Smetanova ulica 17, 2000 Maribor, Slovenia

**Keywords:** Virtual screening, Similarity search, LiSiCA, PyMOL, Molecular graphics

## Abstract

Ligand-based virtual screening of large small-molecule databases is an important step in the early stages of drug development. It is based on the similarity principle and is used to reduce the chemical space of large databases to a manageable size where chosen ligands can be experimentally tested. Ligand-based virtual screening can also be used to identify bioactive molecules with different basic scaffolds compared to already known bioactive molecules, thus having the potential to increase the structural variability of compounds. Here, we present an interface between the popular molecular graphics system PyMOL and the ligand-based virtual screening software LiSiCA available at http://insilab.org/lisica-plugin and demonstrate how this interface can be used in the early stages of drug discovery process.

**Graphical Abstract:**
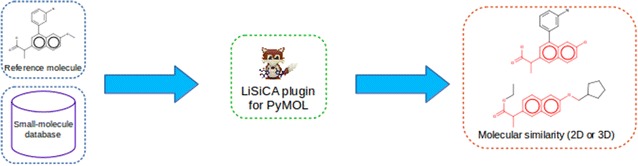
Ligand-based virtual screening interface between PyMOL and LiSiCA.

Ligand-based virtual screening interface between PyMOL and LiSiCA.

## Background

Virtual screening (VS) of large compound libraries has become a standard computational technique in modern drug discovery pipeline [1]. VS is used to search compound libraries to identify structures which are putative binders for a particular drug target. The approach helps in reducing the enormous chemical space of large databases to a manageable size that can be subjected to experimental testing.

Ligand-based virtual screening (LBVS) is one of the two broad approaches of VS, the other being structure-based virtual screening. The central assumption of LBVS is that similar structures have similar biochemical activities [2]. LBVS requires at least one known active ligand that binds to the drug target. The goal of LBVS is to identify molecules with different basic scaffolds but with similar or better biochemical activity compared to the already known bioactive ligands—a concept referred to as scaffold hopping [3].

Efficient visual examination and comparison of predicted ligands is an important part of VS studies, contributing crucially to the choice of compounds that will be subsequently biochemically or biologically evaluated. PyMOL [4] is a widely used molecular graphics program, which has evolved into a platform for several plugins that use its versatile visualization capabilities.

In the field of molecular interactions, several extensively used plugins for PyMOL were developed. The Autodock Vina plugin [5] covers all functionalities of an entire molecular docking workflow, including preparation, execution and analysis of docking input/output files. The APBS plugin [6] is an interface to the adaptive Poisson-Boltzmann solver (APBS) program [7] and enables electrostatics calculations and the visualization of potential energy surfaces and charge densities on protein surfaces. CASTp [8–10] detects pockets and voids in protein structures to determine and characterize binding sites, while Caver [11, 12] performs calculations of substrate pathways and entrance tunnels in protein structures, and enables their visualization.

In this work, we describe a plugin for PyMOL which allows carrying out 2D or 3D ligand-based virtual screenings of large small-molecule databases with immediate subsequent visualization of predicted ligands. The developed plugin represents an interface between PyMOL and LiSiCA, an LBVS software based on a graph theoretical maximum clique algorithm (http://insilab.org/maxclique) [13, 14]. The main purpose of this interface is to enable visualization of large amounts of data returned from LiSiCA’s output within the preferred environment of many researchers. Thus, LBVS becomes straightforward for scientists who may not be comfortable with using the command line interface. The plugin allows specifying the reference ligand and the target database, defining all of the LiSiCA’s options, loading previously completed projects and outputting a list of the highest scoring molecules that can be visualized in the PyMOL viewer. LiSiCA plugin is free for academic use and is available from http://insilab.org/lisica-plugin.

## Implementation

### General constraints

The general requirement for the LiSiCA plugin is an installed PyMOL program (version >1.4) with plugin support. LiSiCA software executable used by the plugin runs under Linux and Windows operating systems. The plugin uses the native Python library Tkinter (https://wiki.python.org/moin/TkInter) and is thus dependent on the underlying Tcl/Tk libraries (version ≥8.5). These, if not installed by default, are available in the package management systems of most Linux distributions; they are installed together with PyMOL in Windows.

### Installation

Standardized PyMOL plugin installation has been adopted for LiSiCA plugin. The user installs the LiSiCA plugin through the PyMOL’s Plugin Manager interface by providing *lisica.py* file downloaded from http://insilab.org/lisica-plugin. When first started, the *lisica.py* script automatically downloads all the remaining required files (executable files, log files, icon files and the python modules) from the remote server to the *.lisicagui* directory located in the user’s home directory, which requires a stable internet connection.

### Plugin graphical user interface

**Fig. 1:**
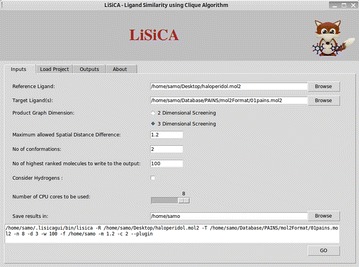
LiSiCA ‘Inputs’ tab, where all the LiSiCA’s options are displayed. In the bottom window the command is displayed that can be used in the command-line to run the standalone executable

The graphical user interface (GUI) for LiSiCA plugin consists of four distinct tabs: ‘Inputs’, ‘Load Project’, ‘Outputs’ and ‘About’ tab (Fig. 1).

### Inputs tab

The ‘Inputs’ tab provides an interface, in which all all the input parameters and options for the virtual screening using the LiSiCA software can be defined. The reference and the target mol2 files are specified in the respective entry widgets and the input validation then checks if the entered mol2 files exist and if they are in the required mol2 format before the execution of the LiSiCA software. Two-dimensional (2D) or three-dimensional (3D) virtual screening can be chosen using the appropriate radio button labeled *product graph dimensions*. The GUI then presents further set of input options according to this choice: for the 2D screening, the *maximum allowed shortest path difference* defined as the maximum difference between the numbers of covalent bonds between two matched atom pairs; for the 3D screening the *maximum allowed spatial distance difference* defined as the tolerance in Ångströms between the coordinates of two matched atom pairs. For both, 2D and 3D screening the user can choose the *number of highest ranking molecules to be outputted*. For 3D screening, the *number of conformations* of a target molecule to be shown in the final output can also be specified. User can also specify if hydrogen atoms in the input ligands should be considered in the generation of the molecular and subsequent product graph. By default, hydrogen atoms are not considered to achieve faster screening.

LiSiCA runs in parallel on multiple CPU cores, thus the plugin allows the user to choose the number of CPU cores to be used. The maximum number of CPU cores available in the system is detected automatically, and by default, all available cores are used.

The location where the results of LiSiCA are to be saved is also specified in the ‘Inputs’ tab. The results files of each LiSiCA run include the text file containing on each line a target molecule’s ID and Tanimoto coefficient expressing its similarity to the reference molecule. Additionally, mol2 files are created, which contain aligned coordinates of the reference and the target molecules’ atoms. Each mol2 file also contains a comment section expressing the common atoms found in both molecules.

An additional feature in the ‘Inputs’ tab is the display of the command that can be entered in the command line for the underlying LiSiCA executable. Potentially, users would prefer to use LiSiCA plugin to reliably create the appropriate screening command and then use the obtained command to run the standalone executable on a large computer cluster.

Once the user sets all the parameters in the ‘Inputs’ tab, the screening can be started by clicking the ‘GO’ button. This initiates the validation of values in all input widgets, followed by the execution of the LiSiCA software in the background. If any inputs are invalid, a dialog box appears with the error or alert message. In case of successful validation, the progress bar indicates that the LiSiCA executable is running. When the process finishes and the output files are written to disk the progress bar is terminated and the ‘Outputs’ tab with the results is automatically opened.

### Load Project tab

The ‘Load Project’ tab allows the user to recover the results and visualization of a previous LiSiCA’s screening run, by selecting the directory containing the output text file and mol2 files. The ‘Load’ button loads all the results in the ‘Outputs’ tab.

### Outputs tab

**Fig. 2:**
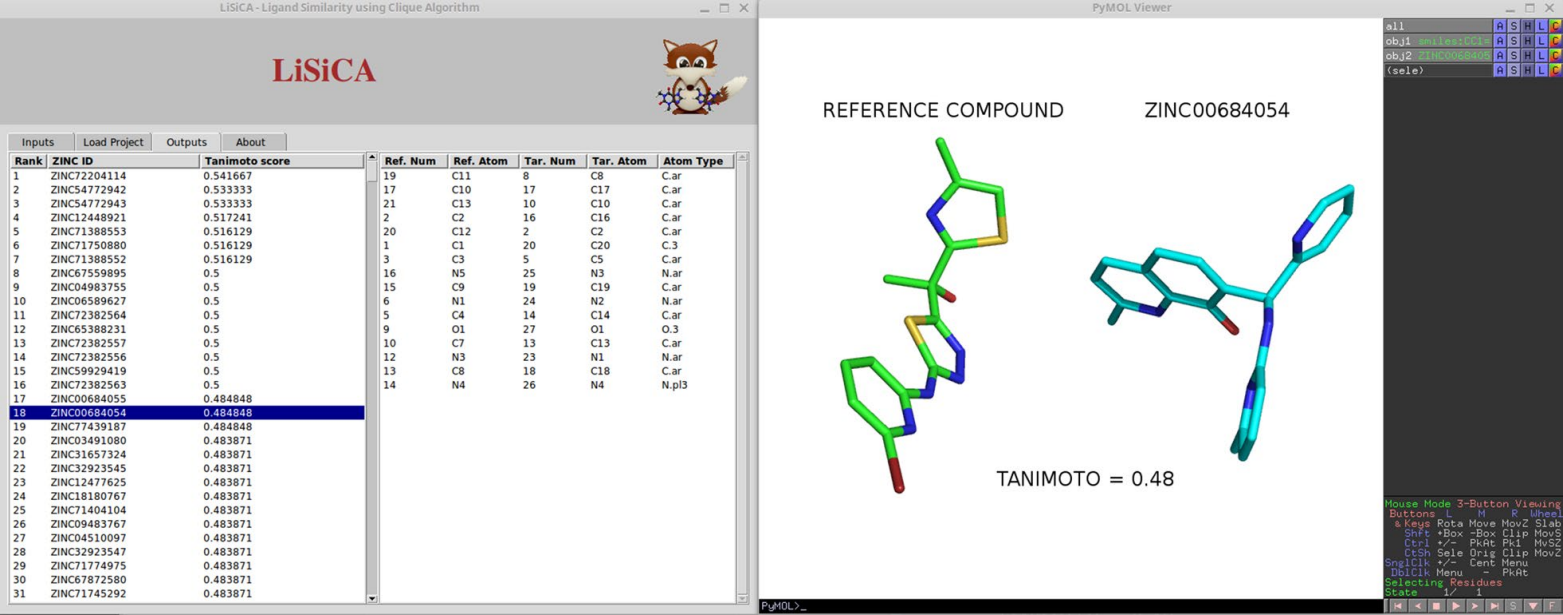
Example of the plugin’s output after 2D screening. On the *left hand side* two lists are shown. The *list on the left* shows the highest scoring target molecules ranked by the Tanimoto coefficient. The currently selected target molecule is displayed in the PyMOL visualizer on the *right side* of the screen (*cyan carbon atoms*), along with the reference molecule (*green carbon atoms*). The second *list on the left hand side* of the screen displays atom pairs (ID number based on the mol2 file and SYBYL atom types) that form the common substructure of the reference and target molecule. The selection of an atom pair can be immediately visualized in the PyMOL visualizer by *purple selection squares* (not shown). The Tanimoto coefficient and the molecule names are displayed for clarity and are not shown in the PyMOL viewer

**Fig. 3:**
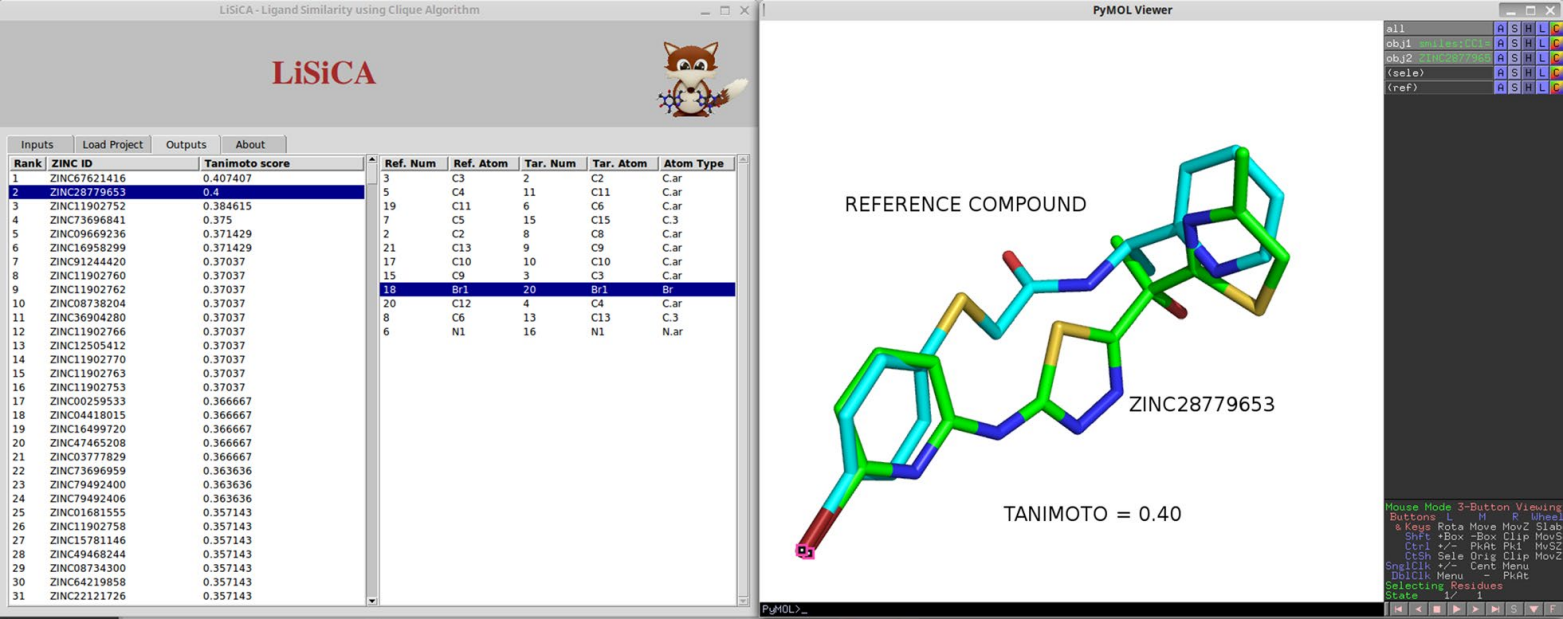
Example of the plugin’s output after 3D screening, where two molecules are superimposed based on the atom pairs displayed on the *left hand side list*. Here, two comparable bromine atoms are selected. *Color codes* are the same as in Fig. 2. The Tanimoto coefficient and the molecule names were added for clarity and are not shown in the PyMOL viewer

The ‘Outputs’ tab presents the LiSiCA’s results in a logical and interactive manner (Figs. 2, 3). The results are presented as two multicolumn lists. On the left hand side of the tab, a multicolumn list is displayed with ranked—according to the decreasing Tanimoto coefficients—target molecules. Each row contains the rank and the name of a target molecule, and its Tanimoto coefficient expressing the similarity between it and the reference compound. By selecting a particular row, the corresponding target molecule is displayed in the PyMOL viewer along with the reference ligand. If 2D screening was performed, the two molecules are displayed side-by-side for easy visual comparison (Fig. 2); in case of the 3D screening, the two molecules are superimposed upon each other using the PyMOL pair fitting function, thus making it possible to visually compare the spatial arrangement of potentially important functional groups (Fig. 3). Moreover, by clicking on a target molecule, the root-mean-square deviation is calculated between the reference and the target matched atoms.

The right hand site list shows the corresponding atom pairs between the currently selected target molecule and the reference molecule. This list contains atom numbers as well as atom types of the matched reference and target molecule atoms that were recognized as similar by LiSiCA. By clicking on a row, the corresponding atom pair is selected and highlighted in the PyMOL viewer, making it easy to identify the common substructures in both compounds.

### About tab

The ‘About’ tab contains the ‘Product Version Information’ section where the LiSiCA GUI version currently used is specified. In the same section, the user can get information on any new updates if available.

## Results and discussion

Here, the LiSiCA plugin was used to screen a known inhibitor of the *Mycobacterium tuberculosis* InhA enzyme [15], an enoyl-acyl carrier protein reductase, against the ZINC Drugs Now database [16] containing approximately 10 million compounds (Fig. 2). InhA is a key enzyme involved in the fatty acid biosynthesis pathway II in the *M. tuberculosis* bacteria. Compared to isoniazid, the first-line drug for the treatment of tuberculosis, which also targets InhA, this active compound does not need preliminary enzymatic transformation with mycobacterial catalase-peroxidase enzyme (KatG) to become active. As KatG mutations are the most common mechanism for isoniazid resistance, this compound and its derivatives are potentially promising candidates for the treatment of infections caused by isoniazid resistant strains. We used the 2D screening option for LiSiCA with all the other options set to default. After screening, which took about an hour to complete on 8 CPU cores, the target compounds were ranked according to their topological similarity to the reference compound expressed by the Tanimoto coefficients. The one hundred highest scoring molecules were visually inspected using the LiSiCA plugin in the PyMOL viewer and the most interesting molecules, that is, ones having different overall scaffolds from the reference ligand, could be purchased from vendors and biochemically tested for their IC50 values. For example, compound ZINC00684054 (Fig. 2) has a very different scaffold compared to the reference molecule, and might, if found active, be a good starting point in a hit to lead procedure; especially as the aromatic rings allow much room for substitution and therefore potential optimization.

### Previous experimental validation of LiSiCA on butyrylcholinesterase enzyme

The LiSiCA software was previously tested for its ability to discriminate active from inactive molecules in the Directory of Useful Decoys Enhanced Database [17] (see Ref. 14). In that study we showed that both the 2D and 3D screening options outperform a competing ShaEP method [18]. With LiSiCA we obtained an average area under the ROC curve of 0.71 ± 0.15 and 0.67 ± 0.16, for 2D and 3D screening respectively, whereas with ShaEP we obtained an average of 0.57 ± 0.11, which is significantly lower compared to the results of both the LiSiCA’s screening options. We also employed LiSiCA for the discovery of new nanomolar butyrylcholinesterase inhibitors [14]; we used the bioactive conformation of a known inhibitor (PDB code: 3F9) [19] obtained from the butyrylcholinesterase enzyme (PDB ID: 4TPK) as a reference molecule and screened it against the ZINC Drugs Now database using the LiSiCA’s 3D option. To cover as much conformational space as possible, we initially prepared on average 188 conformers per each database compound. Finally, the 30 best-ranked ZINC compounds with the highest scoring Tanimoto coefficient were purchased and tested biochemically for their IC50 values: 53 % of the purchased compounds showed >50 % inhibition at 10 μM concentration, while 17 % exhibited inhibition in the nanomolar range (IC50 values ranging from 80 to 840 nM). While none of the nanomolar inhibitors had an IC50 as low as the reference compound (21 nM), all of them exhibited a significantly different molecular structure, and also had higher binding-efficiency indexes compared to the reference molecule. LiSiCA thus proved its ability for obtaining compounds with diverse scaffolds, while the low binding-efficiency index of the structures enables their further optimization.

## Conclusion

We present a novel plugin for the widely used molecular graphics system PyMOL, which allows performing ligand-based virtual screening studies using LiSiCA software. The plugin enables simple preparation of the LiSiCA’s screening procedure, with the ability to customize all the available options for LiSiCA. As visual support is an important aspect in virtual screening, the plugin is expected to enhance the ligand-based virtual screening efforts. In the future, we will extend the plugin with a knowledge-based bioisostere similarity search tool, hopefully furthering the plugin’s usefulness in finding novel active compounds with highly diverse structures.
